# Detorsion is not innocent: pirfenidone prevents contralateral ovarian damage following ovarian torsion

**DOI:** 10.1007/s00210-026-05174-3

**Published:** 2026-03-13

**Authors:** Hamza Halici, Zeynep Karakoy, Emir Enis Yurdgulu, Yusuf Anil Ay, Elif Cadirci, Erdem Toktay, Betul Kavas

**Affiliations:** 1 Department of Hınıs Vocational Training School, Ataturk University, 25100 Erzurum, Turkey; 2 Department of Pharmacology, Faculty of Pharmacy, Erzincan Binali Yıldırım University, Erzincan, Turkey; 3 Department of Biochemistry, Faculty of Pharmacy, Ataturk University, Erzurum, Turkey; 4 Department of Pharmacology, Faculty of Medicine, Ataturk University, Erzurum, Turkey; 5 Department of Histology and Embryology, Faculty of Medicine, Kafkas University, Kars, Turkey

**Keywords:** Ovarian torsion, Ischemia, Reperfusion, Oophorectomy, Pirfenidone

## Abstract

Ovarian torsion is a gynecological emergency caused by rotation of the vascular pedicle, leading to ischemia, necrosis, and potential fertility loss. Management remains controversial, particularly regarding oophorectomy versus ovarian preservation. Detorsed ovaries continue cytokine secretion, impairing contralateral ovarian function, and no protective therapy currently exists. This study aimed to compare oophorectomy and detorsion strategies and to determine whether post-detorsion long-term pirfenidone therapy preserves ovarian function, thereby providing experimental resolution to the clinically pivotal “save or sacrifice” dilemma. Forty female rats were divided into five groups: Healthy, IR, IR + OVX, IR + Pirfenidone 50 mg/kg, and IR + Pirfenidone 100 mg/kg. Ischemia was induced in the left ovary of IR groups; in IR + OVX, the ovary was excised at 3 h, while in other groups, ovaries were reperfused. Treatments continued for 30 days. Ovarian tissues and blood were analyzed histopathologically, biochemically, and molecularly. Ovarian ischemia–reperfusion induced a persistent inflammatory and profibrotic response not only in the ischemic ovary but also in the contralateral ovary, leading to reduced estrogen levels and marked histopathological damage. Importantly, detorsion alone did not prevent contralateral injury, whereas oophorectomy attenuated molecular alterations. Post-detorsion pirfenidone therapy dose-dependently restored estrogen production, suppressed inflammasome activation (IL-1β, NLRP3, caspase-1) and fibrotic signaling (SMAD2, SMAD3, TGF-β1), and preserved normal follicular architecture in both ovaries. These findings demonstrate that the detorsed ovary remains a biologically active source of injury and identify pirfenidone as a potential adjunct therapy capable of protecting ovarian function after torsion.

## Introduction

Ovarian torsion is an emergency gynecological condition affecting women of all ages, though it occurs more frequently during adolescence and requires prompt intervention. In developed countries, it is a recognized cause of infertility. Accurate diagnosis and immediate treatment are crucial to prevent ischemia and necrosis that may lead to functional loss in the affected ovary (Houry and Abbott 2001). Diagnosis relies on the combined evaluation of clinical symptoms and sonographic findings (Moro et al. 2020; Otjen et al. 2020). Risk factors such as prior torsion or ovarian cysts increase suspicion, and once torsion is suspected, surgical intervention is essential (Asfour et al. 2015). In a US emergency department study on adnexal torsion, 758 of 1265 patients were hospitalized, and 263 underwent surgery. Among these, 177 had oophorectomy (67.3%) and 85 underwent minimally invasive procedures (48%) (Das et al. 2024). Thus, treatment can proceed by either oophorectomy or ovarian conservation, and the choice between them remains a matter of clinical debate regarding reproductive outcomes.

Extensive literature supports laparoscopic detorsion over oophorectomy, emphasizing ovarian preservation. Studies have shown that ovarian function can be at least partially restored after detorsion (Asfour et al. 2015; Wei et al. 2018; Sandrieser et al. 2023). Since 2016, the American College of Obstetrics and Gynecology has recommended detorsion and ovarian conservation (2016). However, oophorectomy is still frequently performed in torsion cases (Das et al. 2024).

While detorsion prevents organ loss, ischemia–reperfusion (I/R) injury remains inevitable, as reperfusion induces oxidative stress and inflammation, leading to fibrosis and organ dysfunction (Blaivas and Lyon 2005; Kula et al. 2023). Conversely, oophorectomy decreases fertility potential, as live birth rates with a single ovary are significantly lower than in women retaining both (Lind et al. 2018; Rodriguez-Wallberg et al. 2022). In contrast, laparoscopic detorsion is considered safe, preserving ovarian reserve and antral follicle count without impairing reproductive function (Yasa et al. 2017).

In patients undergoing oophorectomy, various studies and techniques have been developed to preserve the contralateral ovary and maintain fertility. Similarly, different approaches have been described in endometriosis cases to protect the ovaries and/or preserve ovarian reserve (Prapas et al. 2017; Gullo et al. 2020; Laganà et al. 2022; De Paola et al. 2025; Perrone et al. 2025).

The transforming growth factor-beta (TGF-β1)/SMAD pathway plays a major role in fibrosis after I/R injury (Derynck and Zhang 2003; Frangogiannis 2020). Increased TGF-β1 levels have been demonstrated following reperfusion (Wang et al. 2012; Can et al. 2022). Overexpression of TGF-β promotes epithelial–mesenchymal transition (EMT) and extracellular matrix (ECM) accumulation, contributing to fibrotic disorders in the lungs, kidneys, and liver (Peng et al. 2022). This pathway also regulates ovarian physiology (Yu et al. 2016), yet elevated ovarian TGF-β1 levels may cause follicular dysplasia and ovulatory dysfunction (Chu et al. 2018). Inhibition of the TGF-β1/SMAD pathway protects against I/R-induced injury and preserves organ function (Wang et al. 2012; Liu et al. 2023). Therefore, targeting this pathway may prevent fibrosis and restore ovarian function following I/R injury.

Pirfenidone, an FDA-approved antifibrotic agent that inhibits TGF-β and exhibits anti-inflammatory and antioxidant effects, has shown protective outcomes in various organ I/R models (Arumugam et al. 2002; Saito et al. 2019). Pirfenidone has been investigated in various fields due to its regulatory effects on TGF-β signaling pathways, and several studies have reported its significant therapeutic potential. Beneficial effects of pirfenidone have been demonstrated in colonic anastomosis models (Duran et al. 2026), and significant effects in ulcerative colitis have also been reported (Wanas et al. 2026). More recently, studies have documented the effects of pirfenidone in ovarian torsion models (Abdel-Aziz et al. 2025). However, the long-term effects of pirfenidone on ovarian ischemia/reperfusion injury, as well as its potential impact on the contralateral ovary, have not yet been fully elucidated.

This study aimed to compare oophorectomy and detorsion strategies and to determine whether post-detorsion pirfenidone therapy preserves ovarian function, thereby providing experimental resolution to the clinically pivotal “save or sacrifice” dilemma.

## Materials and methods

### Animals

In this study, 40 female Wistar rats (4–5 months old, weighing 250–290 g) were obtained from the Laboratory Animal Center of Ataturk University, Medical and Experimental Practice and Research Center. All animals were housed in plastic cages under standard environmental conditions (22 ± 1 °C, 40–80% relative humidity, and a 12-h light/12-h dark cycle). Throughout the experimental period, all rats had ad libitum access to standard laboratory chow and drinking water. All experimental protocols were conducted in accordance with national guidelines for the care and use of laboratory animals.

### Clinical trial number

Not applicable.

### Ethical statement

This study and all related protocols were approved by the Local Ethics Committee for Animal Experiments of Ataturk University (Meeting Date: 25.12.2024, Meeting Number and Decision No: 2024/13–307).

### Chemicals

Pirfenidone (Pirfect 600 mg, tablet) was obtained from Nobel (Turkey). Xylazine (Xylasinbio, Intermed Drug Warehouse Ltd., Ankara, Turkey) and ketamine (Ketalar, Pfizer, Istanbul, Turkey) were used for anesthesia. All other laboratory-grade chemicals were purchased from Sigma and Merck (Germany).

### Experimental groups

Forty rats were randomly divided into five groups (*n* = 8). Sample size calculation was performed using G Power version 3.1. Based on a two-tailed alpha error of 0.05, a power of 0.8, and an effect size of 0.55, eight rats for each group were calculated as the minimum sample size required to achieve statistical power. Pirfenidone (PIR) in capsule form was powdered and dissolved in distilled water. The PIR doses (50 and 100 mg/kg) were selected based on previous studies (Guo et al. 2019). The experimental groups are summarized in Table 1.

**Table 1:** Experimental groups and treatment protocols

Group	Group name	Application	PIR dose	PIR treatment duration
1	Healthy	Only laparotomy	-	-
2	I/R	3 h ischemia followed by 3 h reperfusion	-	-
3	I/R + OVX	3 h ischemia followed by 3 h reperfusion; subsequently, the left ovary was excised	-	-
4	I/R + PIR 50	After I/R, PIR was administered orally at 50 mg/kg	50 mg/kg	30 days
5	I/R + PIR 100	After I/R, PIR was administered orally at 100 mg/kg	100 mg/kg	30 days

### Surgical procedure for induction of ischemia-reperfusion and oophorectomy

**Fig. 1:**
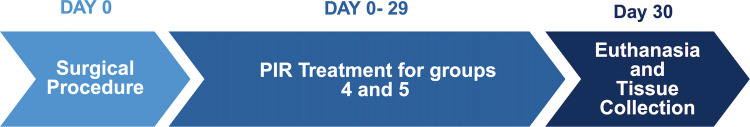
Procedure timeline

Rats were fasted for 12 h before the procedure and anesthetized with an intraperitoneal injection of ketamine (45 mg/kg) and xylazine (5 mg/kg) at the end of the fasting period (Bozkurt et al. 2024). The abdominal area bounded by the costal arch and inguinal region was shaved, disinfected with povidone-iodine, and a lateral 2–3 cm incision was made to access the ovaries through the peritoneum and abdominal muscles. In the Healthy group, only laparotomy was performed without additional intervention. In the ischemia–reperfusion (IR) groups, ischemia was induced in the left ovary using an atraumatic clamp for 3 h, followed by reperfusion for 3 h (Halici et al. 2008). In the IR + OVX group, the ischemic left ovaries were ligated and removed using cautery. In the pirfenidone treatment groups, following ischemia–reperfusion, pirfenidone was administered orally at 50 or 100 mg/kg daily for 30 days. At the end of 30 days, all animals were euthanized under high-dose anesthesia. The right ovaries of the IR + OVX group and both ovaries of the other groups were excised for histopathological and molecular analyses (Fig. 1).

### Biochemical analyses

Serum estrogen levels were analyzed using a commercially available ELISA kit (BT Lab, Shanghai, China) in accordance with the manufacturer’s protocol.

### Molecular analyses

#### RNA isolation from rat ovarian tissue

Ovarian tissues were homogenized using a Tissue Lyser II (Qiagen) with liquid nitrogen and steel beads. RNA isolation was performed with the QiaCube automated system (Qiagen) according to the RNeasy Mini Kit protocol. RNA quantity and purity were determined using a Nanodrop spectrophotometer (EPOCH, BioTek, 260/280 nm).

#### Reverse transcription reaction and cDNA synthesis

cDNA was synthesized from RNA using the High-Capacity cDNA Reverse Transcription Kit (Thermo Fisher, USA). The concentration of cDNA was determined with the EPOCH spectrophotometer (BioTek, Take3 microplate system), and samples were stored at − 20 °C.

#### Quantitative real-time PCR analysis of mRNA expression

mRNA expression of SMAD2, SMAD3, TGF-β, IL-1β, NLRP3, and caspase-1 was quantified, with β-actin serving as the reference gene. Amplification and quantification were performed using a StepOne Plus Real-Time PCR System. Statistical analyses were conducted with GraphPad Prism 10.3.1 software.

### Histopathological and immunohistochemical analyses

#### Tissue processing and hematoxylin and eosin (H&E) staining

Ovarian tissue samples were fixed in 3.7% formaldehyde solution for 72 h. Following fixation, tissues were washed under running water for 30 min to remove formaldehyde. Dehydration was achieved by sequential immersion in graded alcohol solutions (50%, 60%, 70%, 80%, 96%, and 99%) for 1 h each. Tissues were then cleared with xylene (3 × 15 min, 45 min total). Finally, tissues were embedded in paraffin at 60 °C and blocked for sectioning. Paraffin blocks were sectioned at 5 μm thickness using a microtome. Sections mounted on slides were stained with hematoxylin for 5 min and eosin for 2 min. Prepared slides were examined and photographed under an Olympus CX21 microscope with camera attachment. Images were compiled using Photoshop CS5.

#### Immunohistochemical staining method

Sections mounted on adhesive slides were rinsed in distilled water for 5 min, then subjected to heat-induced antigen retrieval in sodium citrate solution under high pressure for 1 min. Slides were washed with phosphate-buffered saline (PBS, pH 7.2) for 5 min and incubated with 3% H2O2 for 10 min to inhibit endogenous peroxidase activity. Non-specific background staining was blocked with a universal protein block for 5 min. Sections were incubated overnight at 4 °C with caspase-3 primary antibodies. The following day, slides were washed three times in PBS (5 min each) and incubated with secondary antibodies for 10 min at room temperature. After further washing, slides were incubated with streptavidin–peroxidase for 10 min and washed again in PBS. 3,3′-Diaminobenzidine (DAB) chromogen was applied for 15 min. Counterstaining was performed with Mayer’s hematoxylin for 2 min, followed by dehydration through alcohol and xylene series, and slides were coverslipped. Immunopositivity was scored as negative (−), mild (+), moderate (+ +), or strong (+ + +).

### Statistical analysis

Statistical analyses were performed using GraphPad Prism 10.3.1. Normality of the data was assessed using both analytical and graphical methods. Real-Time PCR results were evaluated by one-way ANOVA followed by Dunnett’s multiple comparison test. A *p*-value < 0.05 was considered statistically significant. All results were expressed as mean ± standard deviation (SD) for each group.

## Results

### Serum estrogen levels

**Fig. 2:**
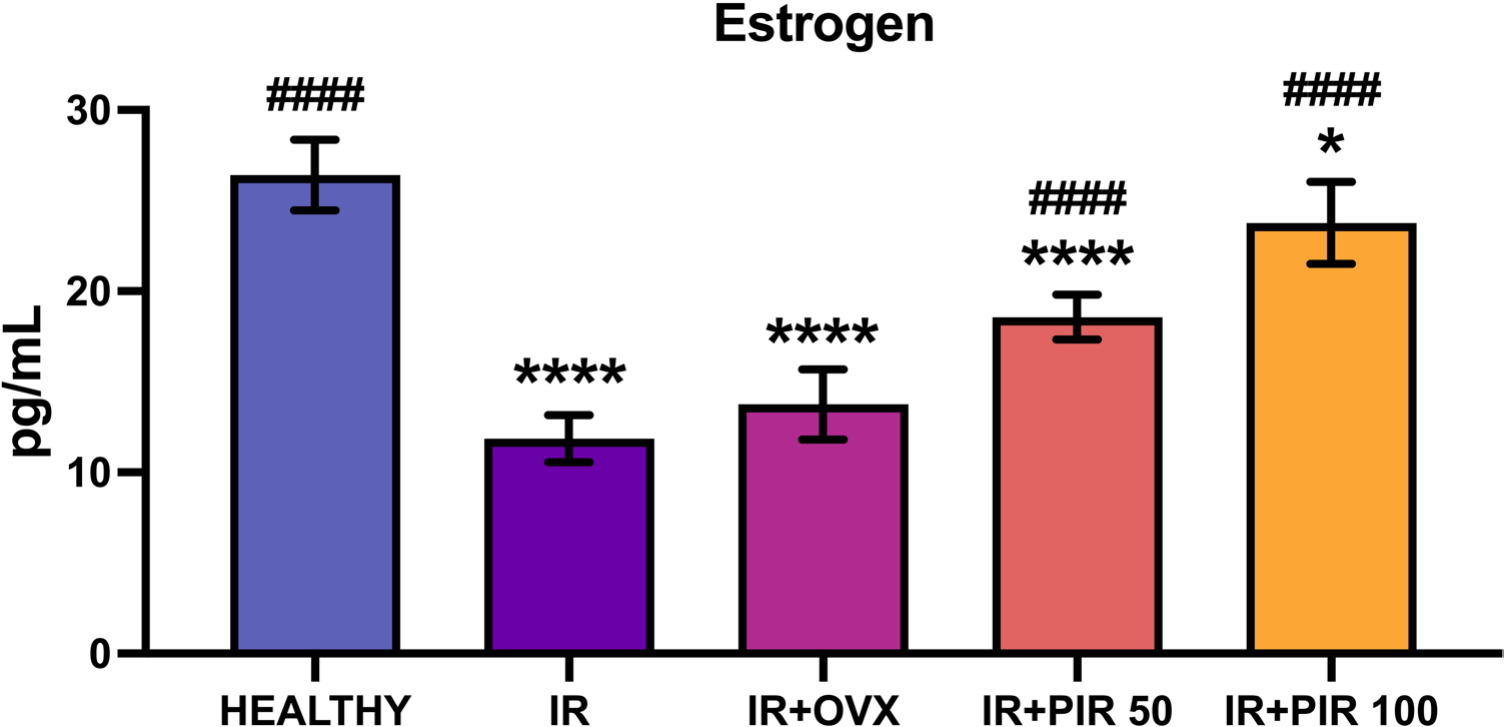
Serum estrogen levels. IR, ischemia/reperfusion; OVX, ooferektomi; PIR50, 50 mg/kg pirfenidone; PIR100, 100 mg/kg pirfenidone. Statistical significance: one-way ANOVA followed by Dunnett’s multiple comparison test. Asterisks (*): comparison vs. Healthy group (*P < 0.05; **P < 0.01; ***P < 0.001; ****P < 0.0001). Hashes (#): comparison vs. IR group (#*P* < 0.05; ##*P* < 0.01; ###*P* < 0.001; ####*P* < 0.0001)

Serum estrogen levels were markedly decreased in the IR group compared with the Healthy group. Interestingly, estrogen levels in the IR group and the oophorectomy group were similarly reduced compared with controls. In the pirfenidone-treated groups, estrogen levels increased in a dose-dependent manner, approaching those of the Healthy group. Pirfenidone significantly reversed the IR-induced reduction in estrogen levels (Fig. 2).

### mRNA expression levels of TGF-β1, SMAD2, SMAD3, IL-1β, NLRP3, and caspase-1 in ovarian tissues

**Fig. 3:**
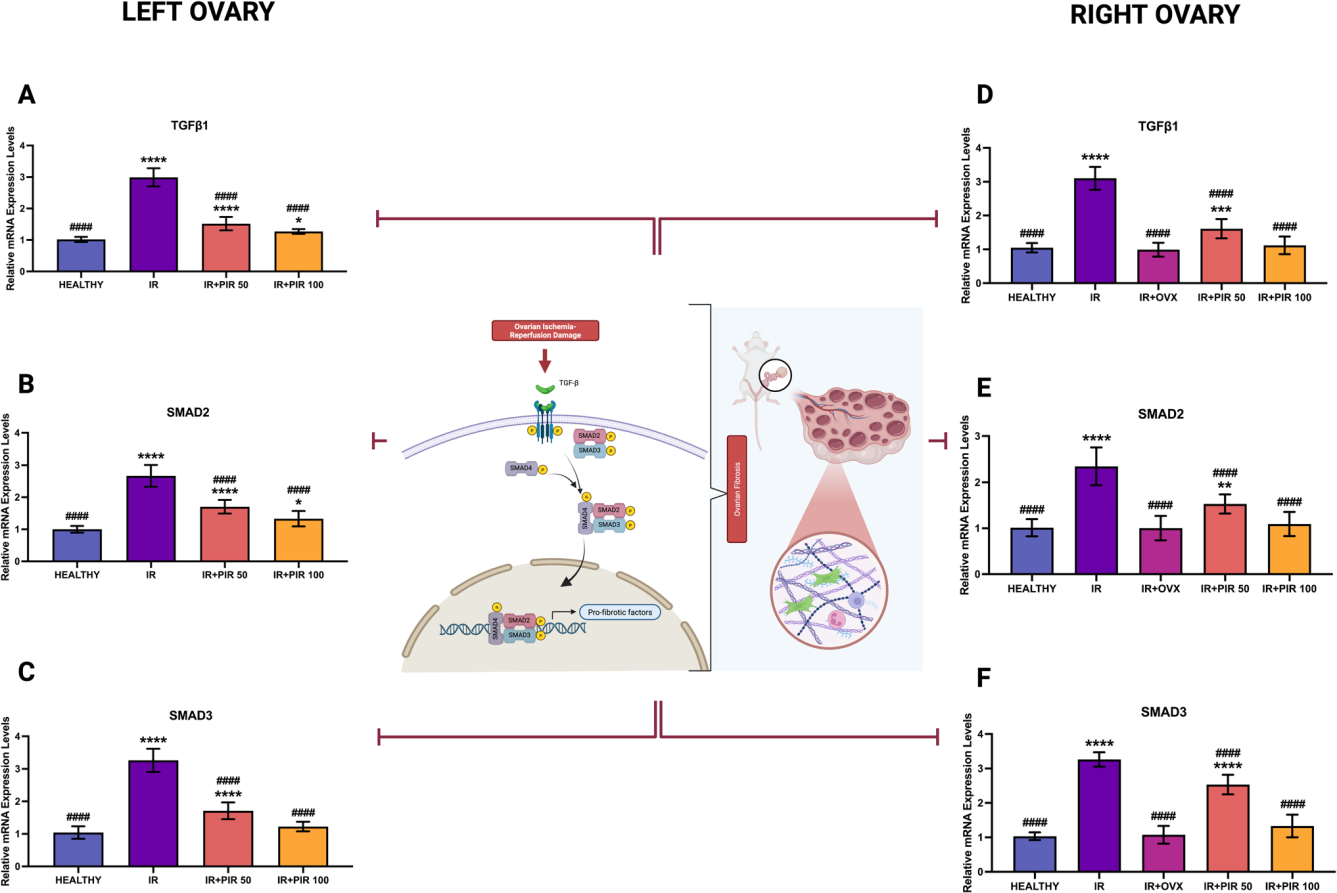
TGFβ1, SMAD2, SMAD3 mRNA expression levels and related pathway. IR, ischemia/reperfusion; PIR50, 50 mg/kg pirfenidone; PIR100, 100 mg/kg pirfenidone. Left ovary: ischemic ovary; right ovary: contralateral ovary. Statistical significance: one-way ANOVA followed by Dunnett’s multiple comparison test. Asterisks (*): comparison vs. Healthy group (*P < 0.05; **P < 0.01; ***P < 0.001; ****P < 0.0001). Hashes (#): comparison vs. IR group (#*P* < 0.05; ##*P* < 0.01; ###*P* < 0.001; ####*P* < 0.0001)

In the contralateral ovary (right ovary) of the IR group, all profibrotic gene expression parameters (TGF-β1, SMAD2, SMAD3) were significantly elevated compared with the Healthy group. In the IR + OVX group, these values were reduced, reaching levels nearly identical to the Healthy group. Similarly, in the groups treated with pirfenidone (50 and 100 mg/kg), expression levels decreased in a dose-dependent manner, resembling the reductions observed in the oophorectomy group (Fig. 3).

**Fig. 4:**
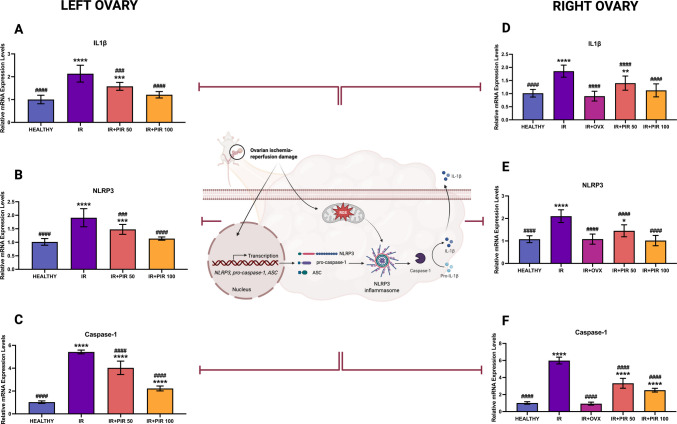
IL1β, NLRP3, caspase-1 mRNA expression levels and related pathway. IR, ischemia/reperfusion; PIR50, 50 mg/kg pirfenidone; PIR100, 100 mg/kg pirfenidone. Left ovary: ischemic ovary; right ovary: contralateral ovary. Statistical significance: one-way ANOVA followed by Dunnett’s multiple comparison test. Asterisks (*): comparison vs. Healthy group (*P < 0.05; **P < 0.01; ***P < 0.001; ****P < 0.0001). Hashes (#): comparison vs. IR group (#*P* < 0.05; ##*P* < 0.01; ###*P* < 0.001; ####*P* < 0.0001)

For inflammasome-related transcripts (IL-1β, NLRP3, caspase-1), IR injury caused a pronounced upregulation of expression. In parallel with the results of profibrotic parameters, expression levels in the oophorectomy group were reduced compared with the IR group. Pirfenidone treatment significantly reversed the strong inflammatory response induced by IR in a dose-dependent manner, restoring expression levels close to those of the Healthy group (Fig. 4).

A similar pattern was observed in the ischemic ovary (left ovary). Ischemia–reperfusion strongly upregulated both profibrotic and inflammasome-related transcripts compared with the Healthy group. Pirfenidone treatment reduced both fibrotic and inflammatory responses in a dose-dependent manner, leading to a significant decrease in expression levels relative to the IR group, approaching those of the Healthy controls (Figs. 3 and 4).

Overall, IR injury consistently upregulated proinflammatory and profibrotic transcripts in both ischemic and contralateral ovaries. Pirfenidone treatment at both doses significantly and dose-dependently normalized these changes.

#### Histopathology and immunohistochemistry results

##### Hematoxylin and eosin (H&E) staining findings

Serial sections of ovarian tissue were carefully examined with respect to cortical and medullary regions. In particular, oocytes and granulosa cells of follicles at different stages of development (primordial, primary, secondary, and Graafian) were evaluated histopathologically.

**Fig. 5:**
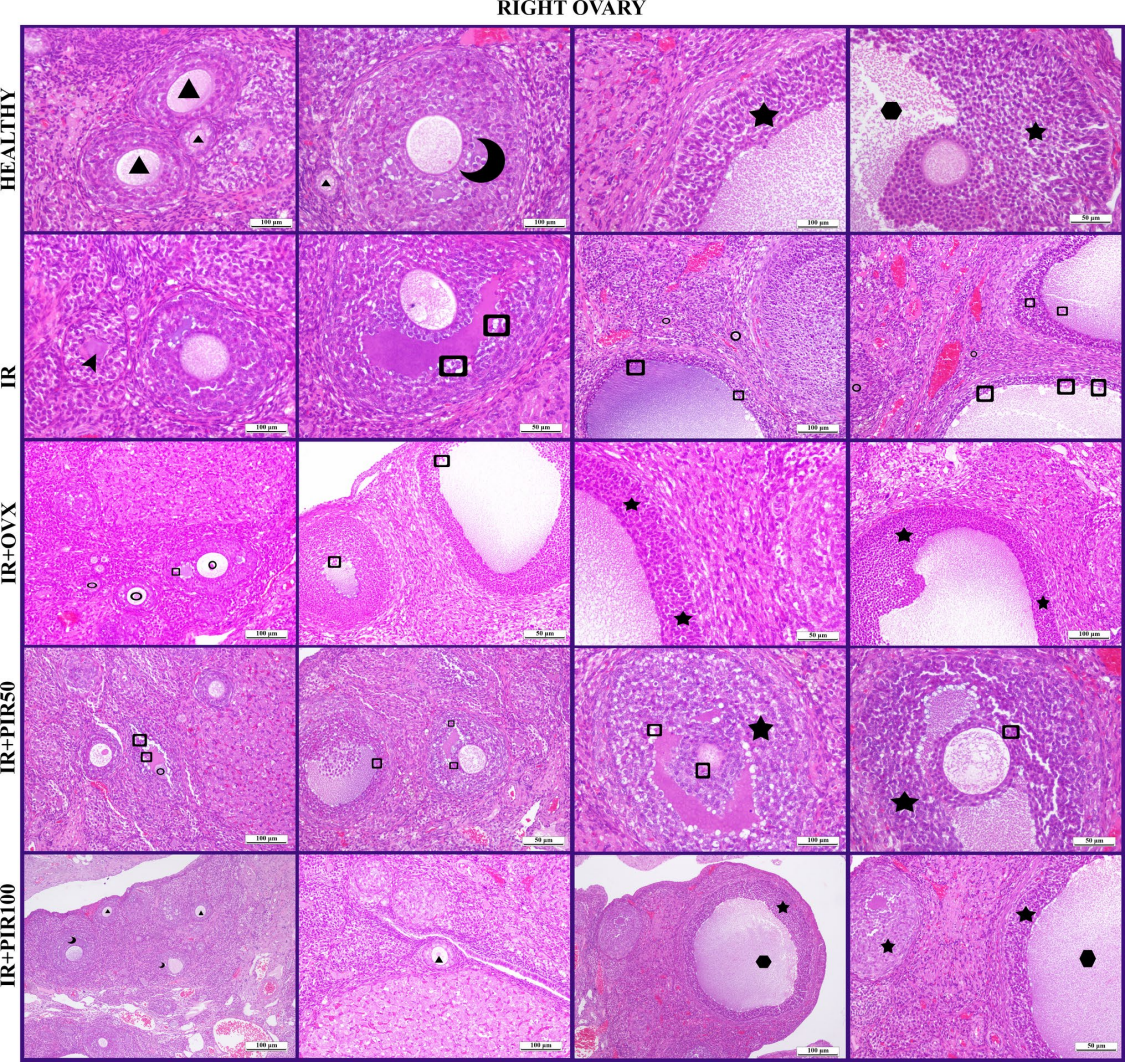
Histopathological findings of the right ovary. Triangle: primary follicle; crescent: secondary follicle; star: granulosa cells; hexagon: Graafian follicle; circle: degenerative oocyte; black square: apoptotic cells; red square: mast cells. E, edema; KS, cystic follicle; IR, ischemia/reperfusion; PIR50, 50 mg/kg pirfenidone; PIR100, 100 mg/kg pirfenidone. Right ovary: contralateral ovary

**Fig. 6:**
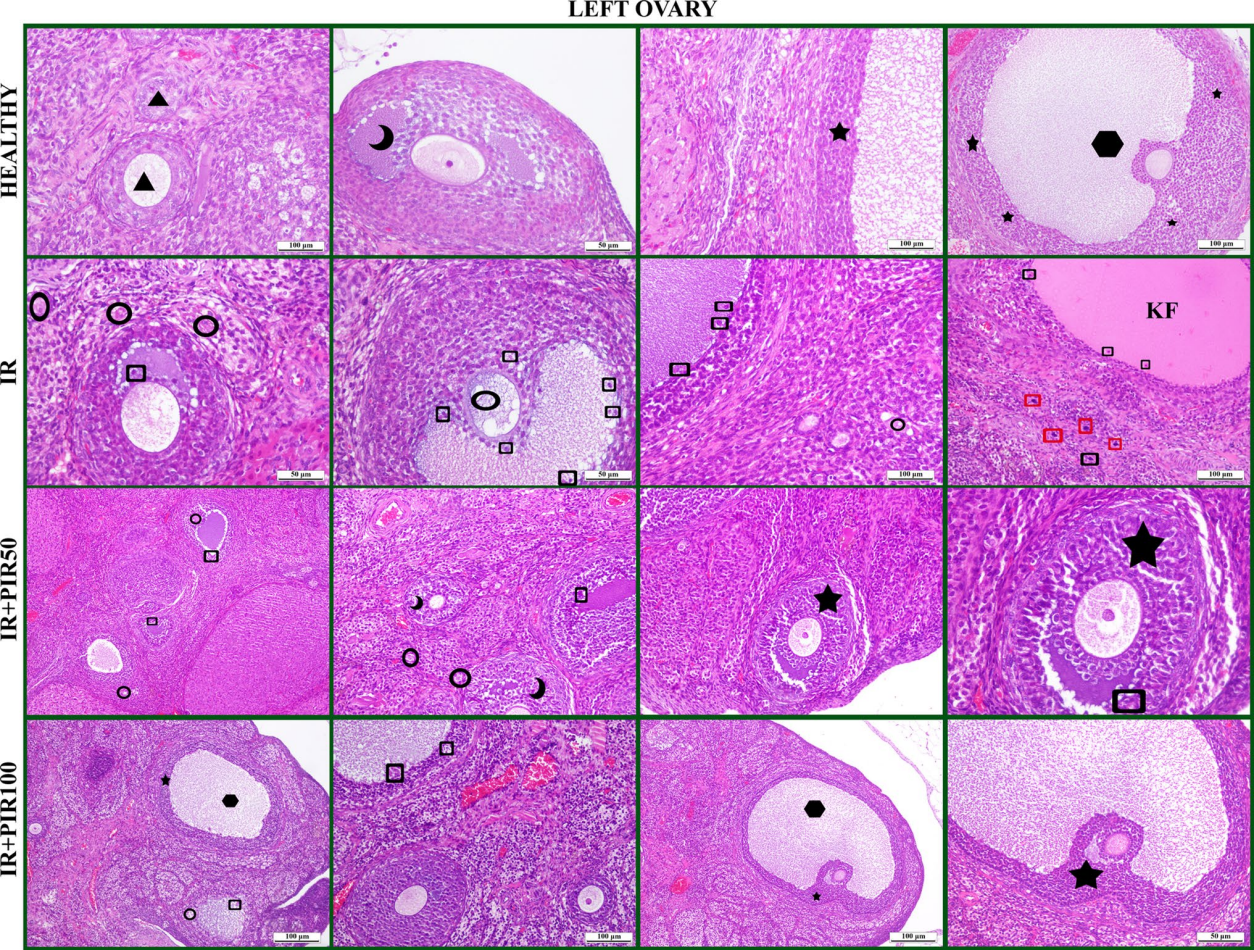
Histopathological findings of the left ovary. Triangle: primary follicle; crescent: secondary follicle; star: granulosa cells; hexagon: Graafian follicle; circle: degenerative oocyte; black square: apoptotic cells; red square: mast cells. E, edema; KS, cystic follicle; IR, ischemia/reperfusion; PIR50, 50 mg/kg pirfenidone; PIR100, 100 mg/kg pirfenidone. Left ovary: ischemic ovary

In the Healthy group, both right and left ovaries displayed follicles at all developmental stages, including primordial, primary, secondary, and Graafian follicles. The granulosa cell layers of secondary and Graafian follicles appeared normal and healthy. Oocytes within follicles exhibited well-defined nuclei and cytoplasm. No pathological findings were observed in this group (Figs. 5 and 6).

In the IR group, the most prominent finding in the right ovary was the transformation of unovulated secondary follicles into corpus luteum, as evidenced by the presence of oocytes within the corpus luteum. Folliculogenesis continued at various stages, but apoptotic granulosa cells were observed in follicles as they grew. When follicles reached the Graafian stage, the granulosa cell layer was markedly thinned. In some primordial follicles, cytoplasmic and nuclear integrity were disrupted, showing clear degeneration. In the left ovary, degenerative follicles were more frequent, with more pronounced apoptotic loss of granulosa cells in the follicular wall. In some Graafian follicles, nearly all granulosa cells were lost, and the follicles transformed into fluid-filled cystic structures. In addition, mast cells were observed within connective tissue of the medulla.

In the IR + OVX group, degenerative follicles and oocytes were not observed in the right ovary. Occasional apoptotic cell loss was noted in some secondary and Graafian follicles. Overall, the histological appearance was similar to that of the Healthy group.

In the IR + PIR50 group, occasional apoptotic granulosa cells were detected in secondary and Graafian follicles. Small degenerative changes were noted in a few early-stage follicles. Similar findings were observed in the left ovary. No mast cells were identified in this group.

In the IR + PIR100 group, follicles at different stages of development in the right ovary exhibited healthy oocytes. Apoptotic loss of granulosa cells in secondary and Graafian follicular walls was almost absent. Similar findings were also observed in the left ovary. No mast cells were detected in this group. The general histological appearance was comparable to that of the Healthy group.

Histopathological findings were semi-quantitatively scored among groups based on the presence of apoptotic cells, degenerative oocytes, and mast cells. Scores were assigned as absent or minimal (−), mild (+), moderate (+ +), or severe (+ + +) (Table 2).

**Table 2:** Scoring of histopathological findings

Groups	Oocyte degeneration		Granulosa cell apoptosis		Mast cell presence	
	Right	Left	Right	Left	Right	Left
Healthy	−	−	−	−	−	−
IR	+	+ + +	+ +	+ + +	−	+
IR + OVX	−	−	+	−	−	−
IR + PIR50	+	+ +	+	+ +	−	−
IR + PIR100	−/+	−	−/+	+	−	−

*IR*, ischemia/reperfusion; *PIR50*, 50 mg/kg pirfenidone; *PIR100*, 100 mg/kg pirfenidone

Left ovary: ischemic ovary; right ovary: contralateral ovary

Absent or minimal (−), mild (+), moderate (+ +), and severe (+ + +)

#### Immunohistochemical findings

Immunohistochemical staining of ovarian tissues was performed using a caspase-3 antibody. Immunopositivity was scored according to staining intensity and the proportion of positive cells: negative (−, 0–10%), mild (+, 10–33%), moderate (+ +, 33–66%), and strong (+ +  +, 66–99%).

**Fig. 7:**
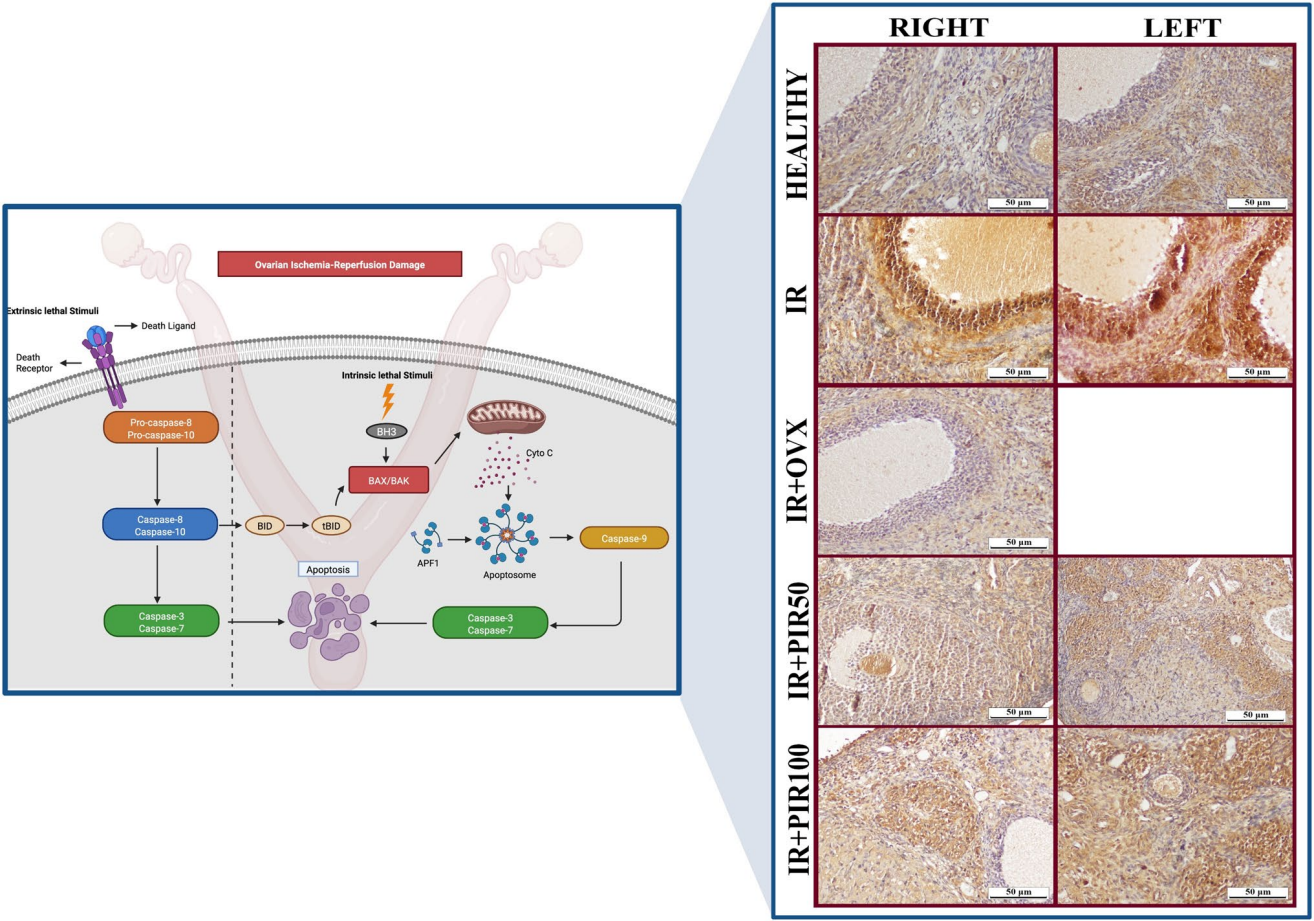
Caspase-3 immunohistochemical staining findings and related pathway. IR, ischemia/reperfusion; PIR50, 50 mg/kg pirfenidone; PIR100, 100 mg/kg pirfenidone. Left ovary: ischemic ovary; right ovary: contralateral ovary

In groups with immunopositivity, caspase-3 staining was prominently localized in granulosa cells of the follicular wall and in luteal granulosa cells of the corpus luteum. In the Healthy group, both right and left ovaries exhibited immunonegativity (−). In the IR group, moderate (+ +) immunopositivity was observed in the right ovary, whereas strong (+ + +) immunopositivity was observed in the left ovary. In the IR + OVX group, immunonegativity (−) was noted in the right ovary. In the IR + PIR50 group, the right ovary exhibited absent to mild (−/+) immunopositivity, while the left ovary showed mild (+) immunopositivity. In the IR + PIR100 group, both ovaries displayed immunonegativity (−) (Fig. 7, Table 3).

**Table 3:** Caspase-3 immunohistochemical staining findings

Groups	Caspase-3	
	Right	Left
Healthy	−	−
IR	+ +	+ + +
IR + OVX	−	−
IR + PIR50	−/+	+
IR + PIR100	−	−

*IR*, ischemia/reperfusion; *PIR50*, 50 mg/kg pirfenidone; *PIR100*, 100 mg/kg pirfenidone

Left ovary: ischemic ovary; right ovary: contralateral ovary

Absent or minimal (−), mild (+), moderate (+ +), and severe (+ + +)

## Discussion

In this study, an ovarian ischemia–reperfusion (IR) model was established in rats to compare the long-term effects of oophorectomy and ovarian detorsion following ovarian torsion, a condition associated with infertility risk. The effects of these two approaches on inflammatory, fibrotic, and apoptotic processes in the ovaries were evaluated using molecular, histopathological, and biochemical analyses. This allowed us to determine which approach caused fewer complications and which was more advantageous in terms of preserving intact ovarian tissue. In addition, we investigated the protective effects of pirfenidone, a TGF-β inhibitor, on both ovaries as a conservative treatment option.

In our study, detorsion of the ischemic left ovary resulted in persistent molecular, biochemical, and histopathological alterations in both the left and contralateral ovaries even after 1 month. In contrast, oophorectomy of the ischemic ovary did not cause such alterations in the contralateral ovary. Notably, in groups that underwent detorsion followed by pirfenidone treatment, the damage in both ovaries was markedly alleviated. In evaluating the contralateral ovaries to determine the relative advantages of oophorectomy versus detorsion combined with pharmacological treatment, we found that long-term pirfenidone administration suppressed the NLRP3–caspase-1–IL-1β and TGF-β–SMAD2/3 pathways after detorsion, while also restoring reduced estrogen levels. These findings were consistent with our histopathological results.

Ovarian torsion, caused by twisting of the vascular pedicle and subsequent disruption of blood flow, is a gynecological emergency that can lead to ischemic necrosis (Bridwell et al. 2022). At the cellular level, hypoxia develops after torsion; during detorsion, reperfusion triggers inflammatory mechanisms through the excessive production of reactive oxygen species (ROS) and activation of the immune response (Ozler et al. 2013; Eser et al. 2015). One of the central mechanisms of inflammation during ischemia–reperfusion injury involves the NLRP3–caspase-1–IL-1β pathway. NLRP3 (NLR family pyrin domain containing 3), a cytosolic sensor protein expressed particularly in macrophages and dendritic cells, functions as a component of the inflammasome (Ghafouri-Fard et al. 2022). Once activated during IR injury, the NLRP3 inflammasome converts pro-caspase-1 into active caspase-1, which subsequently induces IL-1β secretion and inflammation, thereby exacerbating tissue injury (Wu et al. 2022).

Among the cytokines released during IR injury, TGF-β is particularly important. TGF-β regulates multiple cellular processes such as proliferation, differentiation, and extracellular matrix formation, playing a pivotal role in tissue homeostasis (Gu et al. 2014). It is thus one of the most critical cytokines determining organ fate. The TGF-β/SMAD2/3 pathway, a central cascade in inflammation, fibrosis, and tissue remodeling during IR, is activated when TGF-β binds to its membrane receptor, leading to phosphorylation of SMAD2/3. These phosphorylated proteins form a complex with SMAD4, translocate to the nucleus, and activate fibrogenic genes (Chen et al. 2022). It is thus one of the most critical cytokines determining organ fate. The TGF-β/SMAD2/3 pathway, a central cascade in inflammation, fibrosis, and tissue remodeling during IR, is activated when TGF-β binds to its membrane receptor, leading to phosphorylation of SMAD2/3. These phosphorylated proteins form a complex with SMAD4, translocate to the nucleus, and activate fibrogenic genes (Broughton et al. 2009). Mitochondrial stress during ischemia, together with ROS generation and inflammation during reperfusion, triggers mitochondrial disruption and apoptotic pathways (Ulbrich et al. 2014). Released cytochrome c binds Apaf-1 and pro-caspase-9 in the cytoplasm to form the apoptosome, which activates caspase-3. Caspase-3 then degrades cytoskeletal proteins and DNA, culminating in apoptosis (Kim et al. 2005).

In light of this, our study demonstrated that pirfenidone treatment suppressed mRNA expression in the NLRP3–caspase-1–IL-1β pathway. Experimental studies in the literature parallel our findings. Li et al. showed that pirfenidone inhibited NLRP3 inflammasome activation and IL-1β release, thereby attenuating LPS-induced lung inflammation and fibrosis (Li et al. 2018). Similarly, Sharawy and Serrya (2020) demonstrated that pirfenidone improved acute kidney injury by inhibiting the NLRP3 pathway. Consistent with these findings, pirfenidone treatment after detorsion in our study produced significant histopathological improvements and restored estrogen levels. Oophorectomy also prevented excessive expression of the NLRP3–caspase-1–IL-1β pathway in the contralateral ovary, maintaining levels comparable to healthy tissue, as confirmed by our histopathological analyses. By contrast, detorsion alone caused excessive pathway activation and corresponding histopathological damage in both ovaries, consistent with previous studies suggesting that damage in one paired organ can extend to its contralateral counterpart (Bo et al. 2021). Previous studies have demonstrated that the contralateral ovary may also develop injury following unilateral ovarian torsion. However, the underlying mechanism responsible for this phenomenon has not yet been fully elucidated. One study reported that hormonal alterations and changes in blood flow occur in the ovary contralateral to the torsioned ovary (Cakmak et al. 1992, 1994). Similarly, in several paired-organ models, including the adrenal glands and testes, injury to the contralateral organ has also been documented. This injury is thought to be associated with reduced blood flow and hormonal alterations (Nagler and White 1982; Cakmak et al. 1998).

Our study also demonstrated that pirfenidone suppressed detorsion-induced mRNA expression of the critical fibrotic pathway TGF-β–SMAD2/3. Oophorectomy likewise maintained these expression levels at healthy baselines in the contralateral ovary, whereas detorsion alone resulted in significantly elevated mRNA levels compared with controls. This suggests that either post-detorsion pharmacological therapy or oophorectomy may play an important role in preventing fibrotic tissue formation. Moreover, immunohistochemical staining for caspase-3, a key marker of apoptosis, revealed immunonegativity in the oophorectomy group. Improvements were also observed in the pirfenidone-treated groups, indicating that inhibition of inflammatory pathways can limit IR-induced cell death and preserve ovarian tissue integrity. Supporting these findings, Fan et al. reported that pirfenidone ameliorated hepatic IR injury by suppressing increases in TGF-β1 and caspase-3 levels (Fan et al. 2018). However, while oophorectomy did not significantly improve estrogen levels due to tissue loss, pirfenidone treatment after detorsion restored estrogen levels toward those of healthy controls by suppressing inflammatory, fibrotic, and apoptotic pathways.

When reviewing the literature on the “save or sacrifice” dilemma in ovarian torsion, there is no consensus among researchers. Some studies have proposed that paired organs interact, such that pathology in one organ can affect the other (Bo et al. 2021). Indeed, ovarian torsion has been shown to induce both functional and histopathological changes in the contralateral ovary (Cakmak et al. 1993). Circulating free radicals, inflammatory mediators, and cytokines released during IR can cause follicular loss, stromal edema, and apoptotic activation in the intact ovary (Karakoc-Sokmensuer et al. 2016). This risk has led many researchers to advocate oophorectomy as a strategy to eliminate potential contralateral damage. However, oophorectomy carries significant drawbacks, including loss of reproductive potential, disruption of hormonal balance, and long-term health risks (Gasparri et al. 2021; Soh et al. 2022). Consequently, pharmacological treatments are being developed as alternatives to oophorectomy, with the aim of reducing IR injury and preserving ovarian function (Ersoy et al. 2016; Karakaş et al. 2020).

## Conclusion

When the results of our current study are examined, it has been shown that, similar to the literature, even after surgical detorsion following ovarian ischemia–reperfusion, a long-term inflammatory and profibrotic response negatively affecting the ovaries is triggered. These results demonstrate that restoring blood flow through surgery alone does not completely prevent the biological damage. Although oophorectomy alleviates molecular changes on the contralateral side, it cannot be considered a functional solution due to the depletion of ovarian reserve. Conversely, pirfenidone treatment after detorsion successfully inhibited inflammation activation and fibrotic signaling, restored estrogen production, and preserved follicular integrity in both ovaries. Our findings suggest that the detorsed ovary continues to cause damage, that TGF-beta, which controls fibrosis, may play a leading role in this damage, and that additional pharmacological intervention may be necessary after surgical treatment. Therefore, pirfenidone offers a promising treatment approach to preserve ovarian function and may facilitate the transition from purely surgical decision-making to integrated surgical and medical care in cases of ovarian torsion in clinical practice. However, comprehensive future clinical studies are needed to translate these findings into clinical practice.

## Data Availability

The data that support the findings of this study are available from the corresponding author upon reasonable request.
